# Effects of Tea Polyphenols and Microporous Packaging Treatment on Storage and Preservation of Leaf-Vegetable Sweet Potatoes

**DOI:** 10.3390/foods14071191

**Published:** 2025-03-28

**Authors:** Linjiang Pang, Xuefen Lou, Ximing Xu, You Lv, Qingyun Sun, Chengyuan Hu, Yueming Zhu, Xinghua Lu, Chao Xiang, Yuge Guan, Jiyu Cheng, Guoquan Lu, Zunfu Lv

**Affiliations:** 1 Food and Health College, Zhejiang Agriculture and Forestry University, Hangzhou 311300, China; ljpang@zafu.edu.cn (L.P.); lxf3216547jya@163.com (X.L.); ly90313@163.com (Y.L.); xhlu@zafu.edu.cn (X.L.); gyg@zafu.edu.cn (Y.G.); cjy@zafu.edu.cn (J.C.); 2 The Key Laboratory for Quality Improvement of Agricultural Products of Zhejiang Province, College of Advanced Agricultural Sciences, Zhejiang Agriculture and Forestry University, Hangzhou 311300, China; xuximing@zafu.edu.cn (X.X.); qingyun__sun@163.com (Q.S.); huchengyuan@stu.zafu.edu.cn (C.H.); zhuym@zafu.edu.cn (Y.Z.); 3 Institute of Crops and Nuclear Technology Utilization, Zhejiang Academy of Agricultural Sciences, Hangzhou 310021, China; xc0409@126.com

**Keywords:** leaf-vegetable sweet potatoes, tea polyphenols, microporous packaging, fumigation, preservation

## Abstract

Leaf-vegetable sweet potatoes incurred significant losses during storage, which resulted in a shortened shelf life and reduced commercial value. This study investigated the effects of tea polyphenols (TPs) fumigation and microporous packaging (MP) during 10 days at 10 °C and 90–95% RH. The results indicated that the preservation effects followed the order TP + MP > MP > TP > CK, with the TP + MP treatment effectively controlling the degradation rate of chlorophyll and delaying leaf yellowing. In addition, TP + MP treatment increased the activity of antioxidant enzymes, especially catalase (CAT) and ascorbate peroxidase (APX), and also enhanced non-enzymatic systems (flavonoids, total phenolics, and ascorbic acid). Pearson’s correlation analysis showed a positive correlation between the decline in postharvest quality of leaf-vegetable sweet potatoes and the increase in reactive oxygen species (ROS) levels. This study provided a robust theoretical and technical foundation for the development of effective postharvest preservation strategies for leaf-vegetable sweet potatoes.

## 1. Introduction

Among all food commodities globally, fruits and vegetables experience the highest postharvest losses. Due to their high moisture content (typically exceeding 80%), elevated respiration rates, and susceptibility to handling damage, they are particularly prone to postharvest losses and waste [1]. Leaf-vegetable sweet potatoes, which are rich in antioxidant compounds such as phenolics, flavonoids, vitamin C, and carotenoids, are recognized as a highly nutritious vegetable [2]. The Asian Vegetable Research and Development Center has classified them as one of the most nutritionally valuable leafy vegetables [3]. However, due to their large surface area and high moisture content, the leaves suffer from yellowing, rotting, and rapid deterioration after harvest, leading to a short shelf life. Their unique physiology, which includes high metabolic rates and sensitivity to environmental factors such as temperature and humidity, significantly contributes to these postharvest challenges.

To address these challenges, researchers have explored several preservation strategies. Postharvest preservation methods for fruits and vegetables fall into two broad categories: physical and chemical techniques. Physical preservation methods include freezing, irradiation, and other technologies. One study examined the effects of freezing on mushrooms and found that it extended their shelf life while improving their nutritional quality during storage [4]. However, freezing also causes the formation of ice crystals within the food, which affects their size, number, and distribution, ultimately leading to nutrient loss, including proteins, carbohydrates, and fats [5]. In contrast, irradiation methods, such as X-ray and gamma-ray treatments, better preserve the nutritional components of fruits and vegetables, including vitamins and minerals, compared to traditional chemical preservation techniques. However, these methods require expensive equipment, precise dose control, and high technical standards, making them challenging to implement and maintain effectively [6]. Chemical preservation methods traditionally rely on synthetic fungicides, including carbendazim, prochloraz, imazalil, benomyl, and thiabendazole [7]. However, these chemicals have caused various negative effects, such as environmental pollution, health risks to humans, and the emergence of pathogen resistance [8]. As a result, researchers have increasingly shifted toward newer preservation approaches, such as natural preservatives, antibacterial coatings, and modified atmosphere packaging [9]. Most preservation techniques are applied individually, but a single method often proves insufficient. Therefore, integrating multiple technologies and conducting comprehensive research to optimize their combined effects is crucial for enhancing preservation outcomes.

Tea polyphenols (TPs), bioactive compounds naturally found in green tea, exhibit strong antibacterial and antioxidant properties, positioning them as an effective approach to mitigating postharvest deterioration in fruits and vegetables [10]. A key challenge in food preservation is the rapid decline in freshness and nutritional value, primarily caused by oxidation and microbial spoilage. Research indicates that integrating TPs into preservation techniques can significantly alleviate these issues. For instance, oxidation adversely impacts the shelf life and sensory characteristics of fruit juices; however, adding TPs to apple juice has been shown to decelerate oxidative processes, improve antioxidant activity, and sustain flavor stability [11]. Likewise, postharvest mushrooms are prone to enzymatic browning and microbial degradation, but utilizing TP-based packaging films can enhance antioxidant enzyme function, thereby prolonging their quality [12]. Furthermore, seafood such as shrimp deteriorates quickly due to microbial proliferation and oxidative damage. To combat this, scientists have formulated an intelligent TP-infused membrane that not only delays spoilage but also enhances packaging durability, including mechanical strength, UV shielding, and antioxidant efficacy [13]. These advancements underscore the value of TP in addressing critical postharvest preservation challenges and extending the longevity of perishable foods.

Microporous packaging (MP) has been widely recognized as an effective strategy for regulating gas composition within packaging, thereby mitigating anaerobic conditions and preserving the postharvest quality of fruits and vegetables [14]. Improper gas exchange often accelerates spoilage, leading to texture degradation, discoloration, and nutrient loss. MP enhances airflow control, reducing the accumulation of carbon dioxide and preventing oxygen depletion, which are critical factors in maintaining product freshness. For instance, studies have demonstrated that MP, when combined with oxygen scavengers, effectively preserves the firmness and overall quality of fresh strawberries by slowing softening and microbial decay [15]. Similarly, freshly cut broccoli is highly susceptible to yellowing, moisture loss, and glucosinolate degradation, but MP treatment has been shown to significantly delay these processes, thereby extending its shelf life [16]. Moreover, maintaining antioxidant levels in vegetables is essential for preserving their nutritional value, and MP has been found to enhance total polyphenol content and antioxidant activity in cabbage [17]. The effectiveness of MP is largely determined by the size and density of micropores, which regulate the gas exchange rate. By optimizing these parameters, packaging films can be tailored to meet the specific storage requirements of different products [18]. Studies have shown that the increase of CO_2_ and the decrease of O_2_ in the packaging, as well as the changes in the hardness, total pectin, cellulose, and lignin of bitter bamboo shoots, can be significantly inhibited by MP, thereby delaying the aging of bitter bamboo shoots [19]. These findings highlight the potential of MP as a targeted postharvest preservation approach to address the challenges associated with perishable commodities.

Due to their large surface area and high moisture content, leaf-vegetable sweet potato leaves are highly prone to postharvest yellowing, rotting, and rapid deterioration, leading to a significantly shortened shelf life. These issues are largely attributed to their high metabolic activity and sensitivity to environmental conditions, which accelerate quality loss and reduce marketability. To mitigate these challenges, an effective preservation strategy was investigated, combining tea polyphenol (TP) fumigation and microporous packaging (MP). TP, recognized for its strong antibacterial properties, was applied to inhibit microbial growth, while MP was utilized to regulate gas composition and control moisture levels during storage. This integrated approach aimed to establish a controlled microenvironment that would delay yellowing, minimize rotting, and enhance postharvest preservation, thereby addressing the critical issue of rapid deterioration in leaf-vegetable sweet potatoes.

## 2. Materials and Methods

### 2.1. Materials

This study utilized the leafy sweet potato variety “Shulv No. 1” from Jincheng Farm in Lin’an District, Hangzhou. The harvesting method was optimized based on the guidelines provided in Tang et al. [3], with particular attention given to plants that reached 15 cm in length from the growth tip. The harvest typically occurs 90–120 days after planting. Prior to harvesting, both organic and chemical fertilizers were applied to the fields where the leaf-vegetable sweet potato samples were grown. The fertilization strategy primarily involved organic fertilizers, with a smaller quantity of chemical fertilizers used for topdressing. Additionally, nitrogen fertilizers were applied to encourage the rapid growth of stems and leaves. A drip irrigation system was employed to maintain soil moisture without causing waterlogging. During the peak growth period of the stems and leaves, soil moisture content was maintained at approximately 80%, which significantly improved the tenderness of the stem tips.

After ensuring that the samples were free from pests, diseases, and mechanical damage, the selected leaf-vegetable sweet potatoes were promptly transported to the laboratory. Five leaf-vegetable sweet potato plants were selected from each treatment for subsequent research. 

Tea polyphenols (TPs; CAS # 9057-02-07, Shanghai Yuan Ye Biotechnology Co., Ltd., Shanghai, China) were used in this study. A tea polyphenol solution was prepared by dissolving 0.9 g of tea polyphenols in 1 L of distilled water, followed by thorough mixing to ensure homogeneity. Microporous packaging (MP) was created by manually punching holes into polyethylene (PE) fresh-keeping bags. During the preliminary experiment, various concentrations of tea polyphenols (0.3, 0.6, 0.9, and 1.2 g·L^−1^) and different microporous packaging bags with circular hole diameters (1.5 mm with 20 holes, 3 mm with 8 holes, and 6 mm with 16 holes) were tested. Based on the results of the pre-experiment, 0.9 g·L^−1^ tea polyphenols and microporous packaging with 1.5 mm diameter holes and 20 perforations were selected for further study. Four treatment groups of leaf-vegetable sweet potatoes were prepared as follows:

(1) CK: Untreated leaf-vegetable sweet potato samples.
(2) TP: Leaf-vegetable sweet potato samples were placed in an airtight foam box and fumigated with a 0.9 g·L^−1^ tea polyphenol solution by atomization for 30 min.
(3) MP: Leaf-vegetable sweet potato samples were packaged in polyethylene bags with 1.5 mm holes and 20 perforations.
(4) TP + MP: After being fumigated with a 0.9 g·L^−1^ tea polyphenol solution for 30 min, the leaf-vegetable sweet potato samples were naturally air-dried at 25 °C with an airflow rate of 1.5 m/s for 50 min until no visible liquid remained on the leaf surface. Subsequently, the samples were promptly sealed in polyethylene bags with 1.5 mm holes and 20 perforations.

After treatment, the four groups of leaf-vegetable sweet potatoes were placed in an incubator set to a temperature of 10 °C and a relative humidity of 90% for 10 days (simulating the conditions in pre-packaged fruit and vegetable air curtain cabinets in supermarkets). Three replicate samples were randomly selected from each group on days 0, 2, 4, 6, 8, and 10 for further analysis, and the average values of these samples were recorded. Each treatment involved sampling 6 strains of leaf-vegetable sweet potatoes for the determination of weight loss rate, with 3 replicates. For other physiological indicators, 5 strains of leaf-vegetable sweet potatoes were sampled each time, and each index was repeated 3 times.

### 2.2. Headspace Gas Composition Analysis

A gas analyzer (Model 6600, Systech Instruments Ltd., Thame, UK) extracts 6 mL of gas directly from the perforated packaging bag to measure O_2_ and CO_2_ concentrations. The results are expressed as the partial pressure of O_2_ and CO_2_ in %.

### 2.3. Determination of Visual Appearance

The visual appearance of leaf-vegetable sweet potatoes treated with TP, MP, and TP + MP were recorded after 10 days of storage at 10 °C and 90% RH. Leaf yellowing was quantified by measuring color parameters using a colorimeter, while wilting was assessed based on firmness using a texture analyzer.

The firmness of the four sample groups was assessed throughout storage utilizing a TA-type texture analyzer (TMS-PRO, Manufactured by Food Technology Corporation, Great Neck, NY, USA), based on a modified procedure from Zhou et al. [20]. Puncture testing was conducted with a 3.5 mm cylindrical probe at a rate of 10 mm/s. To improve measurement precision, firmness was determined at three separate points along the petiole for each replicate. The results were expressed in Newtons (N).

The color difference (ΔE) among the samples was analyzed using a portable colorimeter (Shenzhen 3nh Tech Co., Ltd., Shenzhen, China). The L*, a*, and b* values represented lightness/darkness, the green-to-red spectrum, and the blue-to-yellow spectrum, respectively. These parameters were utilized to compute ΔE, the overall color difference, following Equation (1), as described by Kim et al. [21].

(1) $${\Delta E}^{*}=\sqrt[{2}]{{\left(L^{*}-{L_{0}}^{*}\right)}^{2}+{\left(a^{*}-{a_{0}}^{*}\right)}^{2}+{\left(b^{*}-{b_{0}}^{*}\right)}^{2}}\;$$

where L_0_*, a_0_*, and b_0_* denote the initial values of untreated leaf-vegetable sweet potatoes prior to storage, while L*, a*, and b* represent the corresponding values for all groups on days 0, 2, 4, 6, 8, and 10 of storage, respectively.

### 2.4. Determination of Weight Loss Rate and Respiration Rate

The weight loss rate and respiration rate of leaf-vegetable sweet potatoes were measured according to the method described by Cai et al. [22]. The respiration rate was monitored within a controlled container using an infrared gas analyzer (HM-GX10, Manufactured by Weifang Hengmei Instrument Co., Weifang, China). The results were quantified in mg/kg per hour.

During the storage period, the weight loss rates of the leaf-vegetable sweet potatoes in each treatment group were monitored at two-day intervals. The weight loss was determined by calculating the difference between the initial and final weights at each measurement interval, following the method described by [23]. The weight loss rate was expressed as a percentage (%) using the formula in Equation (2).

(2) $$Weight\;loss\;rate\;(\%)=\frac{\mathrm{initial}\mathrm{weight}\;-\;\mathrm{final}\mathrm{weight}}{\mathrm{initial}\mathrm{weight}}\times 100\%$$

### 2.5. Determination of Soluble Sugar Content (SSC), Soluble Protein Content, and Chlorophyll Content

SSC was measured using the anthrone reagent method, as described by Pang et al. [24]. A 0.5 mL aliquot of the sample extract was diluted to 1 mL with distilled water. Subsequently, 5 mL of anthrone reagent was added, and the mixture was thoroughly homogenized. The solution was then heated in a boiling water bath for 10 min and rapidly cooled to room temperature. The absorbance was measured at 620 nm, and the sugar content was determined using the standard curve.

The soluble protein content in the leaf-vegetable sweet potatoes was determined according to the procedure outlined by Gu et al. [25], with quantification carried out using bovine serum albumin to construct the standard curve.

Chlorophyll content was assessed following the procedure described by Gu et al. [25]. A 2 g sample was homogenized in 25 mL of 80% acetone, followed by centrifugation at 8000× *g* for 15 min at 4 °C. The supernatant was collected, filtered, and diluted to a final volume of 50 mL using 80% acetone. Absorbance was measured at wavelengths of 663 nm and 645 nm, and the chlorophyll content was calculated using Equation (3).

(3) $$\mathrm{Chlorophyll}\;\mathrm{content}\;(g\cdot {\mathrm{kg}}^{-1})=\frac{\left(20.29\times A_{645nm}+8.05\times A_{663nm}\right)\times V}{m\times 1000}$$

The coefficient “20.29” represents the conversion factor for chlorophyll a at 663 nm, while “8.09” corresponds to the conversion factor for chlorophyll b at 645 nm.

### 2.6. Determination of Malondialdehyde (MDA) Content and Relative Conductivity

MDA content was determined using the thiobarbituric acid (TBA) method described by Huang et al. [26]. The tissue sample was homogenized in 5 mL of 6% (*w*/*v*) trichloroacetic acid (TCA) and then centrifuged at 10,000× *g* for 10 min at 4 °C. The obtained supernatant was used for malondialdehyde (MDA) analysis. A 1 mL portion of the supernatant was mixed with 2 mL of 10% (*w*/*v*) TCA containing 0.5% (*w*/*v*) thiobarbituric acid (TBA). The mixture was subsequently heated to 100 °C for 10 min and then quickly cooled to room temperature. Absorbance was recorded at 450, 532, and 600 nm.

The relative electrical conductivity of leaf-vegetable sweet potato tissues was determined according to the method described in Min et al. [27]. Initially, the samples were punctured and immersed in 20 mL of distilled water for 25 min. The initial electrical conductivity (C_0_) was measured using a conductivity meter (Shanghai Bante Instruments Co., Ltd., Shanghai, China). The distilled water containing the samples was then heated at 100 °C for 5 min to lyse the cells and release all electrolytes. After cooling, the final conductivity (C_1_) was measured. The relative electrical conductivity was calculated using Equation (4).

(4) $$Relative\;conductivity=\frac{c_{1}-\;c_{0}}{c_{0}}\times 100$$

### 2.7. Determination of Superoxide Anion (O_2−_) Generation Rate and Hydrogen Peroxide (H_2_O_2_) Content

The rate of O_2−_ generation was determined according to the method described in Zhou et al. [20]. A 2 g sample was homogenized in 20 mL of 50 mM sodium phosphate buffer (pH 7.8) and subsequently centrifuged at 12,000× *g* for 15 min. The resulting supernatant was collected for the measurement of superoxide production. A 1 mL portion of the supernatant was combined with 1 mL of 50 mM sodium phosphate buffer and 1 mL of 10 mM hydroxylammonium chloride. Following incubation at 25 °C for 20 min, 1 mL of the reaction mixture was mixed with 1 mL of 17 mM 4-aminobenzene sulfonic acid and 1 mL of 7 mM α-naphthylamine. To eliminate pigment interference, the mixture was separated into two phases using ether. Finally, the absorbance of the lower pink aqueous layer was recorded at 530 nm.

The H_2_O_2_ content was measured following the procedure outlined by Xu et al. [27]. A 2 g sample was homogenized in 5 mL of acetone to ensure thorough mixing, preparing it for subsequent analysis, then centrifuged. The supernatant (1 mL) was mixed with acetone (1 mL), 10% titanium tetrachloride hydrochloride (0.1 mL), and ammonia (0.2 mL). The precipitate was washed with acetone and then dissolved in 2 M H_2_SO_4_ (4 mL). Subsequently, the absorbance was measured at a wavelength of 412 nm. 

### 2.8. Determination of Antioxidant-Related Enzyme Activity

The activity of superoxide dismutase (SOD) was measured using the method described by Cai et al. [22]. For SOD activity determination, 0.1 mL of the extracted solution was combined with 3 mL of the SOD reaction mixture, which contained 13 mM nitrotetrazolium blue chloride (NBT), 50 mM phosphate-buffered saline (PBS, pH 7.8), 1.5 µM riboflavin, and 63 µM methionine. The reaction was exposed to 4000 Lux illumination for 30 min and was then immediately halted by placing it in the dark. The absorbance of the resulting solution was measured at 560 nm. One unit of SOD activity was defined as the amount of enzyme required to inhibit 50% of NBT photoreduction per minute.

Peroxidase (POD) activity was determined using a modified method based on EI-Saber Batiha et al. [28]. A 2 g sample was homogenized in an extraction buffer, and the resulting mixture was centrifuged at 10,000× *g* for 15 min at 4 °C. The supernatant was then collected and used for enzyme assays. POD activity was defined as the amount of enzyme required to cause a 0.01 change in absorbance at 470 nm per minute, per gram of tissue, per milliliter of the reaction system.

Catalase (CAT) activity was measured using a modified version of the method described by Pang et al. [24]. To obtain the supernatant, a 2 g sample was homogenized with 1 mL of extraction reagent on ice and then subjected to centrifugation at 4542× *g* for 10 min at 4 °C. The collected supernatant (35 μL, pH 7) was subsequently mixed with a reaction mixture containing 50 mM phosphate buffer and 15 mM H_2_O_2_. The change in absorbance at 240 nm over a 1 min period was used to determine the rate of H_2_O_2_ decomposition. One unit of catalase (CAT) activity was defined as the amount of enzyme required to break down 1 μmol of H_2_O_2_ per minute per gram of tissue. The final results were expressed as U·g^−1^.

The activity of ascorbate peroxidase (APX) was measured using a modified method based on the procedure described by Xu et al. [29]. The reaction mixture for APX activity determination (3 mL) contained 0.5 mM ascorbic acid, 0.1 mM EDTA-2Na, and 0.1 mM H_2_O_2_, all dissolved in 0.05 M sodium phosphate buffer (pH 7.0), along with 0.1 mL of the enzyme solution. The decrease in absorbance at 290 nm was monitored, which indicated the oxidation of ascorbic acid. One unit of enzyme activity was defined as the oxidation of 1 µmol of ascorbic acid per minute at 290 nm.

### 2.9. Determination of Flavonoid Content, Total Phenol Content (TPC), and Ascorbic Acid (ASA)

The determination of total phenolic content (TPC) and flavonoids was conducted using a modified method based on the procedure outlined in Gong et al. [30]. A 2 g sample was mixed with quartz sand and ground in an ice bath with 3 mL of a 1% HCl-methanol solution. The extraction process was carried out at 4 °C in the dark for 2 h with continuous shaking. Following extraction, the mixture was centrifuged at 8000× *g* for 10 min, and the resulting supernatant was collected for analysis. Gallic acid served as the standard for TPC determination, with absorbance measured at 280 nm to generate the standard curve. The total phenolic content was expressed as the gallic acid equivalent (g·kg^−1^) per gram of sample. For flavonoid quantification, rutin was used as the standard, and its standard curve was established based on absorbance at 325 nm. The total flavonoid content was reported as rutin equivalent (g·kg^−1^) per gram of sample.

The determination of ASA content followed the method described in Sang et al. [31]. Initially, 2 g of the sample was homogenized with 100 mL of 2% oxalic acid, then subjected to centrifugation at 12,000× *g* for 10 min at 4 °C. Subsequently, 1 mL of the obtained supernatant was transferred into a conical flask and titrated with a standardized 2,6-dichlorophenolindophenol solution, with the volume consumed carefully recorded. An oxalic acid solution was used as a blank control in place of the filtrate. The final results were expressed as g·kg^−1^.

### 2.10. Statistical Analysis

The experimental data were analyzed using SPSS 27.0 software. Significant differences were assessed using Duncan’s test, with a significance level of *p* < 0.05. Results are presented as mean values along with their corresponding standard deviations. Furthermore, correlation plots were generated using Origin Pro 2021 software (OriginLab Corporation, Northampton, MA, USA).

## 3. Results

### 3.1. Gas Composition in Different Perforated Packaging Bags

**Figure 1:**
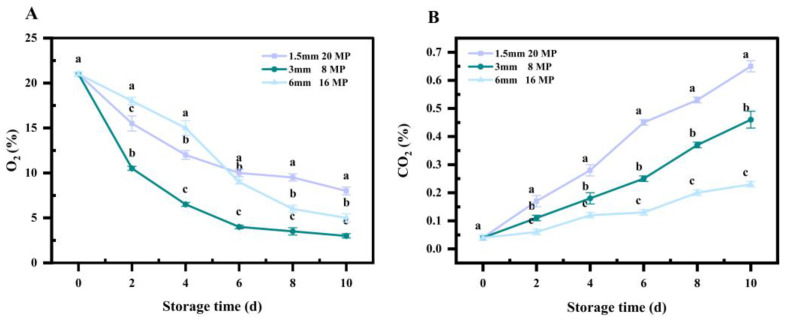
O_2_ (**A**) and CO_2_ (**B**) levels in leaf-vegetable sweet potatoes treated with 1.5 mm, 20 MP; 3 mm, 8 MP; and 6 mm, 16 MP after 10 days of storage at 10 °C and 90% RH. Data are presented as means ± standard errors, based on three replicates. Vertical bars represent standard errors of means. Statistically significant differences (*p* < 0.05) are indicated by different lowercase letters.

As shown in Figure 1, a rapid decline in O_2_ content was observed in all three types of packaging, indicating oxygen consumption due to vegetable respiration. Among them, the O_2_ concentration in the 3 mm, 8 MP decreased the most, suggesting that the gas permeability was lower, leading to faster oxygen depletion. Throughout the storage period, the O_2_ level in the 1.5 mm, 20 MP declined at a slower rate. Meanwhile, the CO_2_ concentration in all three types of bags increased over time, demonstrating continuous CO_2_ release from respiration. Notably, the CO_2_ accumulation in the 1.5 mm, 20 MP was significantly higher than in the other groups. During the entire storage period, the CO_2_ concentration ranged from 0.04% to 0.65% in the 1.5 mm, 20 MP, from 0.04% to 0.46% in the 3 mm, 8 MP, and from 0.04% to 0.23% in the 6 mm, 16 MP. In conclusion, the 1.5 mm, 20 MP was selected for subsequent experiments based on its ability to regulate gas exchange effectively.

### 3.2. Effects of Different Treatments on Visual Appearance

**Figure 2:**
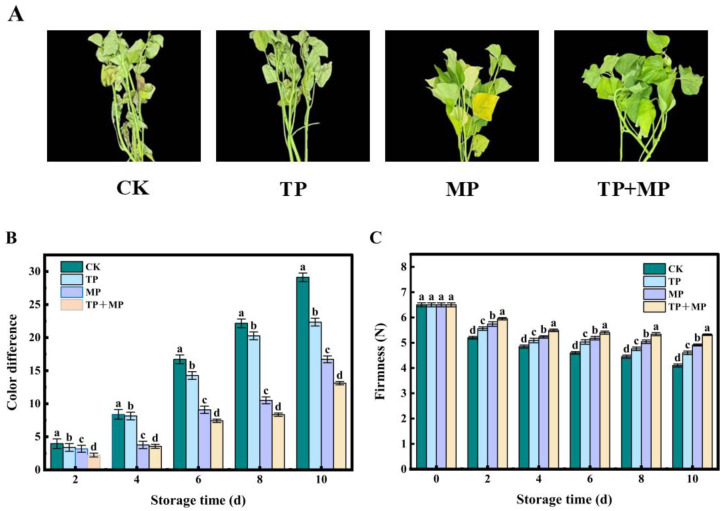
The visual appearance (**A**), color difference (**B**), and firmness (**C**) of leaf-vegetable sweet potatoes treated with TP, MP, and TP + MP were compared with the control group after 10 days of storage at 10 °C and 90% RH. The data are expressed as means ± standard errors, based on three replicates. The vertical bars indicate the standard errors of the means. Statistically significant differences (*p* < 0.05) are denoted by different lowercase letters.

As shown in Figure 2A, the extent of surface browning and wilting in leaf-vegetable sweet potatoes progressively intensified as the storage period lengthened. After 10 days of storage, the CK group exhibited noticeable wilting, characterized by water loss and yellowing of the leaves. In contrast, all treatment groups displayed less severe wilting, with the TP + MP group demonstrating the best overall appearance. This treatment significantly delayed leaf yellowing and effectively preserved leaf color.

As depicted in Figure 2B, the ΔE value for the CK group continuously increased throughout the storage period. In contrast, the ΔE values for all treatment groups remained significantly lower than those of the CK group. Among the treatments, the TP + MP group exhibited the smallest ΔE in both the stems and leaves of the leaf-vegetable sweet potatoes. As storage time progressed, the firmness of the leaf-vegetable sweet potatoes declined in all groups, including the control group (Figure 2C). After 10 days of storage, the control group displayed the lowest firmness (4.1 N), followed by the TP group (4.6 N), the MP group (4.91 N), and the TP + MP group, which retained the highest firmness (5.32 N). These results indicate that the combined TP + MP treatment significantly slows the loss of firmness in leaf-vegetable sweet potatoes.

### 3.3. Effects of Different Treatments on Weight Loss and Respiration Rate

**Figure 3:**
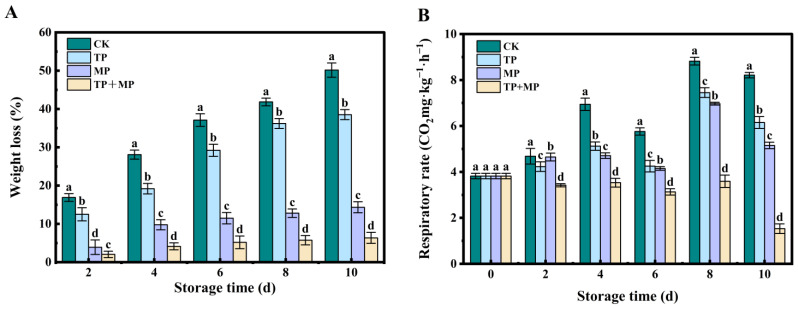
The weight loss (**A**) and respiration rates (**B**) of leaf-vegetable sweet potatoes treated with TP, MP, and TP + MP were compared with the control group after 10 days of storage at 10 °C and 90% RH. The data are expressed as means ± standard errors, based on three replicates. The vertical bars indicate the standard errors of the means. Statistically significant differences (*p* < 0.05) are denoted by different lowercase letters.

The weight loss of the leaf-vegetable sweet potatoes steadily increased as storage time progressed (Figure 3A). After 10 days, all treatment groups showed a significant reduction in weight loss compared to the CK group, with statistically significant differences observed between the treatments (*p* < 0.05). The TP + MP group displayed the lowest weight loss rate.

Throughout the storage period, the respiration rate of leaf-vegetable sweet potatoes in all groups initially increased before gradually decreasing (Figure 3B). Notably, the TP + MP group exhibited significantly lower respiration rates compared to the control and other treatment groups, suggesting that the TP + MP treatment effectively reduces respiration during storage.

### 3.4. Effects of Different Treatments on Soluble Protein Content, Soluble Sugar Content (SSC), and Chlorophyll Content

**Figure 4:**
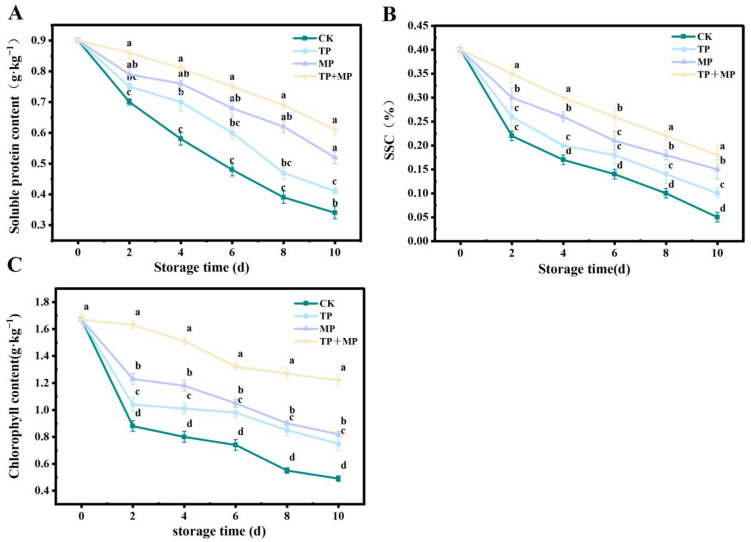
The soluble protein content (**A**), soluble sugar (**B**), and chlorophyll content (**C**) of leaf-vegetable sweet potatoes treated with TP, MP, and TP + MP were compared with the control group after 10 days of storage at 10 °C and 90% RH. The data are expressed as means ± standard errors, based on three replicates. The vertical bars indicate the standard errors of the means. Statistically significant differences (*p* < 0.05) are denoted by different lowercase letters.

As illustrated in Figure 4A, the soluble protein content in all treatment groups declined over the storage period. Significant differences (*p* < 0.05) were observed between the treatments. Notably, after 10 days of storage, the TP + MP group maintained the highest soluble protein content, whereas the CK group exhibited the lowest levels.

The SSC of leaf-vegetable sweet potatoes is shown in Figure 4B. The SSC mass fraction was initially 0.4% at the beginning of storage. After 10 days, the content decreased to 0.05% in the CK group, 0.1% in the TP group, 0.15% in the MP group, and 0.18% in the TP + MP group. The decrease in SSC was slower in the TP + MP group compared to the other treatments.

As shown in Figure 4C, all treatment groups maintained higher chlorophyll content compared to the CK group. After 10 days of storage, the chlorophyll content in the CK group declined to only 0.49 g·kg^−1^, while the TP + MP group retained the highest level at 1.22 g·kg^−1^. These results suggest that the TP + MP treatment effectively slowed the degradation of chlorophyll.

### 3.5. Effects of Different Treatments on Malondialdehyde (MDA) Content and Relative Conductivity

**Figure 5:**
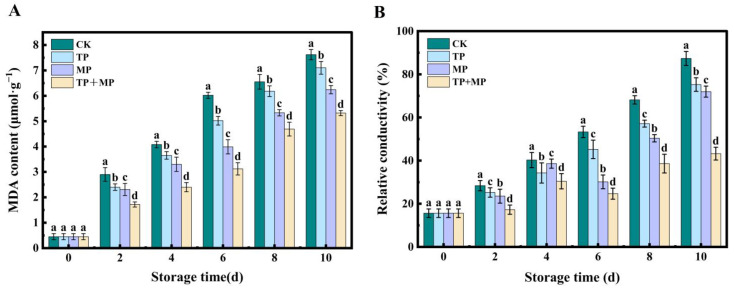
The malondialdehyde (MDA) content (**A**) and relative conductivity (**B**) of leaf-vegetable sweet potatoes treated with TP, MP, and TP + MP were compared with the control group after 10 days of storage at 10 °C and 90% RH. The data are expressed as means ± standard errors, based on three replicates. The vertical bars indicate the standard errors of the means. Statistically significant differences (*p* < 0.05) are denoted by different lowercase letters.

As illustrated in Figure 5A, the MDA content in the CK group increased rapidly throughout the storage period, while the TP, MP, and TP + MP groups exhibited a more gradual rise. Notably, after 10 days of storage, the TP + MP group demonstrated the lowest MDA content, indicating that the combined TP + MP treatment was the most effective in mitigating MDA accumulation.

As shown in Figure 5B, the relative conductivity of all groups increased throughout the storage period. However, the treated groups (TP, MP, and TP + MP) exhibited a significantly slower increase in relative conductivity compared to the CK group. After 10 days, the TP + MP group recorded the highest relative conductivity at 87.36%, while the CK group showed the lowest value at 43.25%. Additionally, a statistically significant difference (*p* < 0.05) was observed between the TP + MP group and the other groups.

### 3.6. Effects of Different Treatments on Superoxide Anion (O_2_^·−^) Generation Rate and Hydrogen Peroxide (H_2_O_2_) Content

**Figure 6:**
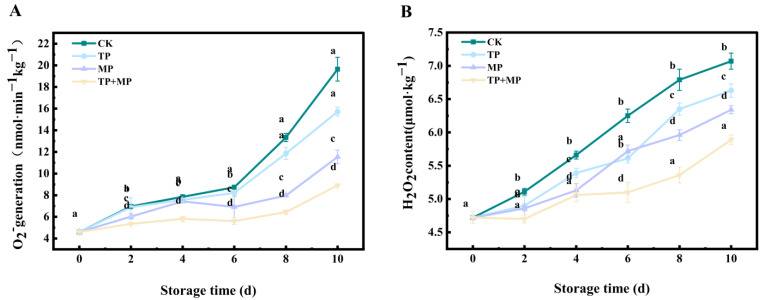
The superoxide anion (O_2_^−^) generation rate (**A**) and hydrogen peroxide (H_2_O_2_) content (**B**) of leaf-vegetable sweet potatoes treated with TP, MP, and TP + MP were compared with the control group after 10 days of storage at 10 °C and 90% RH. The data are expressed as means ± standard errors, based on three replicates. The vertical bars indicate the standard errors of the means. Statistically significant differences (*p* < 0.05) are denoted by different lowercase letters.

Throughout the storage period, the O_2_^·−^ generation rate increased in all groups (Figure 6A), with the CK group consistently exhibiting the highest levels. Notably, after 10 days of storage, the TP + MP group demonstrated a significantly lower O_2_^−^ generation rate compared to the other treatments (*p* < 0.05). Similarly, the H_2_O_2_ content steadily increased over time (Figure 6B). After 10 days, it was evident that the TP, MP, and TP + MP treatments effectively minimized the accumulation of reactive oxygen species (ROS) in comparison to the CK group.

### 3.7. Effects of Different Treatments on APX, CAT, POD, and SOD Activity

**Figure 7:**
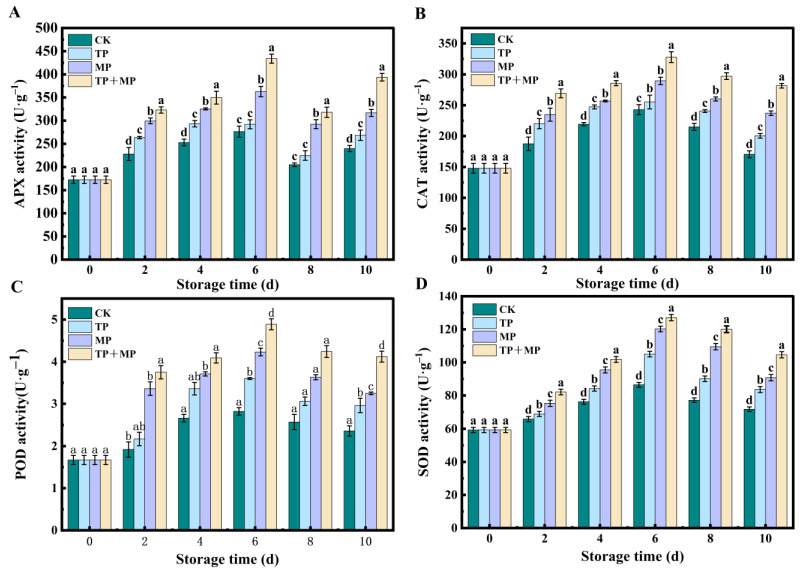
The APX (**A**), CAT (**B**), POD (**C**), and SOD (**D**) activity of leaf-vegetable sweet potatoes treated with TP, MP, and TP + MP were compared with the control group after 10 days of storage at 10 °C and 90% RH. The data are expressed as means ± standard errors, based on three replicates. The vertical bars indicate the standard errors of the means. Statistically significant differences (*p* < 0.05) are denoted by different lowercase letters.

As illustrated in Figure 7A, the APX activity in all groups initially increased and then gradually declined over the storage period. Peak APX activity was reached on day 6 in all groups, with the TP + MP group showing significantly higher activity (433.93 U·g^−1^) compared to the other treatments (*p* < 0.05). After 10 days of storage, APX activity remained elevated in the CK, TP, MP, and TP + MP groups to varying extents. Among these, the TP + MP group recorded the highest APX activity at 393.93 U·g^−1^.

The CAT activity of leaf-vegetable sweet potatoes under different treatments is presented in Figure 7B. In all groups, CAT activity increased from day 0 to day 6, followed by a gradual decline. During the storage period, the CAT activity in the treated groups consistently surpassed that of the CK group. After 10 days of storage, the TP + MP group exhibited the highest CAT activity at 281.52 U·g^−1^, followed by the MP group at 237.2 U·g^−1^, the TP group at 200.48 U·g^−1^, and the CK group at 170.56 U·g^−1^.

Figure 7C shows that the POD activity increased in all groups during the first 6 days, followed by a gradual decline. Throughout the 9-day storage period, the TP + MP group consistently exhibited the highest POD activity at 4.12 U·g^−1^, while the CK group showed the lowest activity at 2.36 U·g^−1^. This suggests that the TP + MP treatment effectively enhanced POD activity to some extent.

All groups exhibited an initial rise in SOD activity from day 0 to day 6, after which a gradual decrease was observed (Figure 7D). Throughout the storage period, the SOD activity in the treatment groups consistently exceeded that of the CK group, and this trend remained evident over the 10-day storage period. Among the treatments, the TP + MP combination exhibited the highest SOD activity, reaching 104.59 U·g^−1^, which was significantly higher than that of the other groups (*p* < 0.05). These results indicate that the TP + MP treatment effectively enhanced SOD activity during storage.

### 3.8. Effects of Different Treatments on Total Phenol Content (TPC), Flavonoid Content, and Ascorbic Acid (ASA)

**Figure 8:**
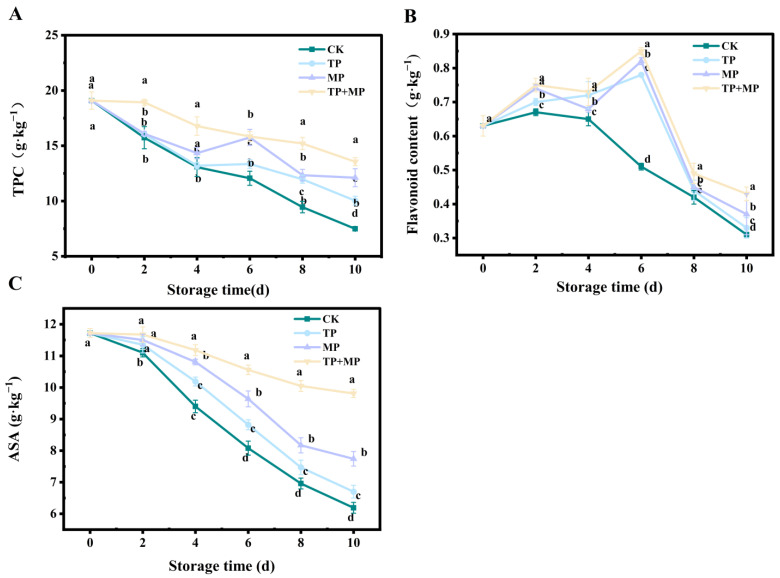
The total phenol content (TPC) (**A**), flavonoid content (**B**), and ASA levels (**C**) of leaf-vegetable sweet potatoes treated with TP, MP, and TP + MP were compared with the control group after 10 days of storage at 10 °C and 90% RH. The data are expressed as means ± standard errors, based on three replicates. The vertical bars indicate the standard errors of the means. Statistically significant differences (*p* < 0.05) are denoted by different lowercase letters.

From day 0 to day 10, the total phenolics content of control leaf-vegetable sweet potatoes showed a continuous decreasing trend. When compared to the CK group, the treatment groups exhibited significantly higher TPC levels (*p* < 0.05). Notably, the TP + MP group maintained the highest TPC after 10 days of storage, outperforming the other groups (Figure 8A).

As can be seen in Figure 8B, the flavonoid content in each treatment initially increased, followed by a gradual decline. The peak flavonoid content occurred on day 6 for all groups, with the TP + MP group maintaining the highest level at 0.85 g·kg^−1^. In comparison, the flavonoid contents of the TP, MP, and CK treatments were 0.78 g·kg^−1^, 0.82 g·kg^−1^, and 0.51 g·kg^−1^, respectively. Notably, after 10 days of storage, the TP + MP group continued to exhibit the highest flavonoid content, significantly outperforming the other treatments.

The ASA levels in all groups decreased over the course of storage. After 10 days (Figure 8C), the TP + MP group exhibited the highest ASA level at 0.18 mg·kg^−1^, followed by the MP group at 0.15 mg·kg^−1^, the TP group at 0.10 mg·kg^−1^, and the CK group, which had the lowest level at 0.05 mg·kg^−1^. These results suggest that the TP + MP group effectively delayed the degradation of ASA during storage.

### 3.9. Correlation Analysis

**Figure 9:**
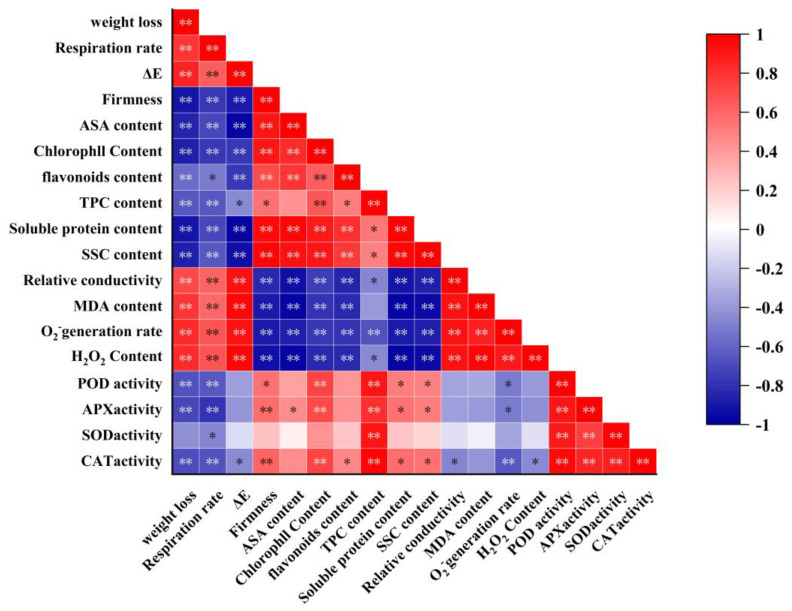
Heatmap of Pearson’s correlations based on measured parameters in leaf-vegetable sweet potatoes after 10 days of storage at 10 °C and 90%RH. (*) indicates significant differences between postharvest treatment indicators. Asterisk (*) indicates significant correlation at *p* < 0.05, and double asterisk (**) indicates significant correlation at *p* < 0.01.

The Pearson’s correlation analysis shown in Figure 9 reveals both positive and negative correlations between the different parameters assessed in this study. Weight loss was significantly and positively correlated with MDA, H_2_O_2_ content, O_2_^−^ generation rate, respiration rate, relative conductivity, and ΔE (*p* < 0.01). Additionally, hardness, chlorophyll content, SSC, soluble protein, and TPC were positively correlated with antioxidant enzyme activities (POD, SOD, APX, and CAT), and negatively correlated with ΔE, MDA, H_2_O_2_ content, and O_2_^−^ generation rate. Furthermore, hardness, ASA, SSC, and soluble protein content were significantly and positively correlated with antioxidant enzymes (POD, CAT, and APX) (*p* < 0.05). In conclusion, the senescence of leaf-vegetable sweet potatoes during storage is driven by elevated ROS levels, which lead to a decline in overall quality.

## 4. Discussion

Postharvest water loss was a key factor affecting the quality of fresh produce, as it could initiate fruit shriveling and deterioration [32]. The leaves of leaf-vegetable sweet potatoes had a large surface area and contained a high amount of water. Once harvested, they were highly susceptible to rapid respiration and decay. In this study, TP, MP, and TP + MP treatments were found to delay weight loss and reduce respiration rates, while the TP + MP treatment showed the most pronounced effects. The findings of Zhang et al. [33] in celery and Raffo et al. [34] in arugula are consistent with the results obtained in this study. Notably, TP or MP treatments also reduced the respiration rate of leaf-vegetable sweet potatoes, which may have been attributed to MP ability to optimize the gas composition within the packaging, creating a microenvironment conducive to slowing respiration. Additionally, the TP + MP treatment proved effective in slowing the reduction of SSC, firmness, and soluble protein levels. This effect may have been due to the regulatory role of MP in gas composition, which significantly delayed macromolecule degradation and reduced nutrient consumption caused by respiration in the later stages of storage. Chlorophyll, a key pigment in photosynthesis, along with carotenoids and anthocyanins, played an essential role in the coloration of fruits and vegetables [32]. The most evident characteristic observed during the storage of leaf-vegetable sweet potatoes was leaf yellowing, caused by chlorophyll degradation. Previous research indicated that microporous packaging significantly delayed chlorophyll degradation. Our research showed that the TP + MP treatment effectively delayed chlorophyll degradation, thereby maintaining the leaves’ bright green color during 10 days of storage. This effect may have been related to reduced activity of chlorophyll-degrading enzymes and the suppressed expression of related genes. As illustrated in Figure 1, the untreated leaves in the CK group wilted and rotted, while those in the TP and MP groups exhibited varying degrees of browning and wilting. In contrast, the TP + MP group maintained the best appearance, which may have been attributed to the antibacterial properties of TP. TP likely disrupted cell permeability, causing metabolic disorders that led to bacterial death. Consequently, fumigating leaf-vegetable sweet potatoes with TP prior to storage effectively resisted external microbial contamination.

The accumulation of ROS during storage led to oxidative damage and lipid peroxidation of plant cell membranes [35]. During postharvest storage, fruits and vegetables underwent gradual senescence, resulting in the accumulation of ROS that disrupted cellular metabolism. The findings of this study revealed that the TP, MP, and TP + MP treatments significantly lowered O_2_^−^ and H_2_O_2_ levels, mitigated the increase in relative conductivity, and reduced MDA content. These findings suggested that TP had a protective effect against oxidative damage to lipids and proteins that constituted cell membranes. As is consistent with these results, previous studies showed that under room temperature storage conditions, the membrane permeability of litchi [36] treated with TP remained minimal. As a result, the integrity of the cell membrane was preserved. Additionally, studies on MP treatment in postharvest winter jujube revealed that MP effectively suppressed the rise in MDA content and minimized oxidative damage induced by ROS [14], aligning with the findings of this study.

To mitigate oxidative damage caused by ROS, the plant tissues relied on two primary mechanisms: enzymatic and non-enzymatic antioxidant systems [37]. Among these POD, SOD, CAT, and APX played critical roles in scavenging ROS in fruits and vegetables [35]. In this study, significant enhancement of antioxidant enzyme activity, including POD, SOD, CAT, and APX, was observed following TP and MP treatments, suggesting that excessive ROS production was alleviated by increased enzymatic function. It was demonstrated that polyphenols activate ARE-regulated genes through transcription factors such as Nrf2, resulting in the upregulation of genes involved in antioxidant enzyme synthesis and consequently strengthening cellular defense against oxidative stress [38]. Additionally, O_2_ and CO_2_ levels within the package are modulated by MP treatment, thereby reducing oxidative stress caused by hyperoxic conditions. Under MP conditions, ROS generation is limited due to decreased oxygen availability, contributing to a reduction in oxidative damage. Moreover, it was suggested that modifications in gas composition activate hypoxia-responsive transcription factors, such as ERF, which are known to play roles in stress adaptation and ROS scavenging [39]. The observed increase in antioxidant enzyme activity is inferred to have contributed to maintaining the quality of leaf-vegetable sweet potatoes, delaying leaf yellowing and wilting. These findings are consistent with previous research, in which TP or MP applications, particularly in combination with other preservation techniques, were shown to enhance antioxidant enzyme activity. For instance, TP incorporation into chitosan- or zein-based composite membranes was reported to suppress peroxidase and polyphenol oxidase activity while concurrently increasing antioxidant enzyme function [12]. Notably, among all treatments, TP + MP consistently resulted in the highest antioxidant enzyme activity in leaf-vegetable sweet potatoes. 

In addition to the enzymatic antioxidant system, non-enzymatic antioxidant systems also played a crucial role in preventing oxidative damage in fruits and vegetables. The non-enzymatic antioxidant mechanism in plants heavily relied on a range of intrinsic antioxidant compounds, including TPC, flavonoids, and ASA. Together, these compounds formed an essential defense system against oxidative stress in plant tissues [40]. This study revealed that TP treatment effectively slowed the reduction in phenolic content, a result is consistent with the findings reported by [11], who reported that the degradation rate of total phenolics in apple products fortified with green tea was significantly slower than in standard apple products. Additionally, the ASA content in the TP + MP group remained consistently higher throughout the storage period compared to the CK group and other treatment groups. This effect was likely attributable to the competitive inhibition of ascorbic acid oxidation by tea polyphenols or the enhancement of ascorbic acid stability through interactions with these compounds [41]. Similarly, TP and MP treatments promoted flavonoid accumulation in leaf-vegetable sweet potatoes, likely due to the TP + MP treatment, which increased flavonoid levels during storage and thereby enhanced the antioxidant capacity. This finding was consistent with results from studies on the use of aloe vera gel and MP treatment in jujube fruit [42]. 

Pearson’s correlation analysis further demonstrated a strong association between reactive oxygen metabolism and the deterioration of leaf-vegetable sweet potatoes quality. Specifically, the decline in leaf-vegetable sweet potato quality after harvest was accompanied by increased weight loss, respiration rate, and ROS levels. In this study, TP treatment was found to reduce the respiration rate of postharvest leaf-vegetable sweet potatoes, thereby influencing their physiological metabolism and alleviating oxidative damage caused by increased ROS levels. MP treatment modified the gas environment within the packaging, potentially impacting the activity and expression of antioxidant enzymes in leaf-vegetable sweet potatoes, which in turn enhanced their antioxidant capacity. Both TP and MP treatments were effective in reducing oxidative damage to varying extents, thereby maintaining the overall quality of the sweet potatoes. Notably, the TP + MP treatment demonstrated superior effectiveness in preserving the quality of leaf-vegetable sweet potatoes. 

## 5. Conclusions

In summary, this study demonstrated the effectiveness of TP, MP, and TP + MP treatments in preserving postharvest leaf-vegetable sweet potatoes. Among them, the TP + MP treatment exhibited the most significant preservation effect, suggesting that it was the most effective approach within the scope of this study. The research results showed that the TP + MP treatment delayed the yellowing of vegetable sweet potato leaves primarily by inhibiting chlorophyll degradation and enhancing the antioxidant system. However, the preservation effect of the TP + MP treatment on other fruits and vegetables may differ, and its regulatory mechanism needs to be further explored through metabolomics, transcriptomics, and other methods in the future. Additionally, the application of TP + MP preservation technology on various fruits and vegetables warrants further investigation to identify more effective preservation methods.

## Data Availability

The original contributions presented in this study are included in the article. Further inquiries can be directed to the corresponding authors.

## References

[1] Marçal S., Sousa A.S., Taofiq O., Antunes F., Morais A.M., Freitas A.C., Barros L., Ferreira I.C., Pintado M. (2021). Technology, Impact of postharvest preservation methods on nutritional value and bioactive properties of mushrooms. Trends Food Sci. Technol..

[2] Jang Y., Koh E. (2019). Antioxidant content and activity in leaves and petioles of six sweet potato (*Ipomoea batatas* L.) and antioxidant properties of blanched leaves. Food Sci. Biotechnol..

[3] Tang C.-C., Ameen A., Fang B.-P., Liao M.-H., Chen J.-Y., Huang L.-F., Zou H.-D., Wang Z.-Y. (2021). Nutritional composition and health benefits of leaf-vegetable sweet potato in South China. J. Food Compos. Anal..

[4] Min D., Dong L., Shu P., Cui X., Zhang X., Li F. (2018). The application of carbon dioxide and 1-methylcyclopropene to maintain fruit quality of ‘Niuxin’ persimmon during storage. Sci. Hortic..

[5] Aghajani P.F., Firouz M.S., Chamgordani P.A. (2023). The improvement of freezing time and functional quality of frozen mushrooms by application of probe-type power ultrasound. Ultrason. Sonochemistry.

[6] Odueke O.B., Farag K.W., Baines R.N., Chadd S.A. (2016). Irradiation Applications in Dairy Products: A Review. Food Bioprocess Technol..

[7] Khan S., Aked J., Magan N. (2001). Control of the anthracnose pathogen of banana (*Colletotrichum musae*) using antioxidants alone and in combination with thiabendazole or imazalil. Plant Pathol..

[8] Wang T., Song Y., Lai L., Fang D., Li W., Cao F., Su E. (2024). Sustaining freshness: Critical review of physiological and biochemical transformations and storage techniques in postharvest bananas. Food Packag. Shelf Life.

[9] Ribeiro I.S., Maciel G.M., Bortolini D.G., Fernandes I.D.A.A., Maroldi W.V., Pedro A.C., Rubio F.T.V., Haminiuk C.W.I. (2024). Sustainable innovations in edible films and coatings: An overview. Trends Food Sci. Technol..

[10] Ma M., Gu M., Zhang S., Yuan Y. (2024). Effect of tea polyphenols on chitosan packaging for food preservation: Physicochemical properties, bioactivity, and nutrition. Int. J. Biol. Macromol..

[11] Zhong W., Yuan W., Wang J., Wu Z., Du H., Huang X., Liu Y. (2024). Antioxidant and preservation effects of tea polyphenols on apple juice. Food Biosci..

[12] Wang X., Huang X., Zhang F., Hou F., Yi F., Sun X., Yang Q., Han X., Liu Z. (2022). Characterization of chitosan/zein composite film combined with tea polyphenol and its application on postharvest quality improvement of mushroom (*Lyophyllum decastes* Sing.). Food Packag. Shelf Life.

[13] Chen S., Zhang H., Jiang Z., Ding X., Chen W., Ma N., Xu S., Yang L. (2024). Intelligent active packaging of sodium alginate and pectin mixed with Aronia melanocarpa anthocyanins and tea polyphenols for shrimp freshness monitoring and preservation. Int. J. Biol. Macromol..

[14] Zuo Z., Jiang P., Chen D., Zhang C., Guo F., Nie X., Wu D., Fan X., Zhao H. (2024). Improving the storage quality and antioxidant capacity of postharvest winter jujube by laser microporous modified atmosphere packaging. Sci. Hortic..

[15] Zhang H., Huang S., Zhao Y., Tian H.S., Lin M., Xie Y., Yu Z. (2025). Modification of microporous bionanocomposite films with visible light-activated photocatalytic antimicrobial TNT-CuO nanoparticles for active fruit packaging. Food Res. Int..

[16] Huie J., Suqiu Z., Haiyan J., Zhijian L., Lijuan C., Nihao L., Xinhua L. (2023). Microporous chitosan/polyvinyl alcohol based active packaging materials with integrated gas-transmission, radiation-cooling, anti-microbial, and ultraviolet shielding features. Chem. Eng. J..

[17] Yuan S., Zuo J., Li X., Fan X., Li X., Wang Q., Zheng S. (2021). Micro-perforated packaging delays leaf yellowing and maintains flavor of postharvest pak choi (*Brassica rapa* subsp. chinensis) following low-temperature storage. Food Packag. Shelf Life.

[18] Chen X.-M., Mou Z.-L., Zhao Y.-T., Su X.-G., Han Y.-C., Chen H.-J., Wei W., Shan W., Kuang J.-F., Lu W.-J. (2024). Modified atmosphere packaging maintains stem quality of Chinese flowering cabbage by restraining postharvest lignification and ROS accumulation. Food Chem. X.

[19] Wang L., Zhang S., Luo Z., Chen Y., Qi Y., Ye M., Chen F., Huang H., Dai F. (2025). Combined modified atmosphere package and melatonin treatments delay the senescence of bitter bamboo shoots by inhibiting the cell wall changes after harvest. LWT.

[20] Zhou Q., Ma C., Cheng S., Wei B., Liu X., Ji S. (2014). Changes in antioxidative metabolism accompanying pitting development in stored blueberry fruit. Postharvest Biol. Technol..

[21] Kim D.-H., Kim H.-B., Chung H.-S., Moon K.-D. (2014). Browning control of fresh-cut lettuce by phytoncide treatment. Food Chem..

[22] Cai S., Zhang Z., Wang J., Fu Y., Zhang Z., Khan M.R., Cong X. (2024). Effect of exogenous melatonin on postharvest storage quality of passion fruit through antioxidant metabolism. LWT.

[23] Li M., Wang M., Hu S., Sun J., Zhu M., Ni Y., Wang J. (2022). Advanced Coatings with Antioxidant and Antibacterial Activity for Kumquat Preservation. Foods.

[24] Pang X., Huang Y., Xiao N., Wang Q., Feng B., Ali Shad M. (2024). Effect of EVA film and chitosan coating on quality and physicochemical characteristics of mango fruit during postharvest storage. Food Chem. X.

[25] Gu S., Fu L., Ren H., Wang X., Huang J. (2019). Effects of exogenous cholesterol treatment on quality characteristics of pak choy (*Brassica chinensis* L.) during storage. Postharvest Biol. Technol..

[26] Huang J., Sun R., Cao X., Hu N., Xia B., Yi Y., Zhou S., Zhou H. (2023). Preservation effect of Lactobacillus plantarum O_2_ fermentation supernatant on postharvest pepper and its induced resistance to Phytophthora capsici. Plant Physiol. Biochem..

[27] Xu F., Zhang K., Liu S. (2020). Evaluation of 1-methylcyclopropene (1-MCP) and low temperature conditioning (LTC) to control brown of Huangguan pears. Sci. Hortic..

[28] El-Saber Batiha G., Hussein D.E., Algammal A.M., George T.T., Jeandet P., Al-Snafi A.E., Tiwari A., Pagnossa J.P., Lima C.M., Thorat N.D. (2021). Application of natural antimicrobials in food preservation: Recent views. Food Control.

[29] Xu D., Zuo J., Li P., Yan Z., Gao L., Wang Q., Jiang A. (2020). Effect of methyl jasmonate on the quality of harvested broccoli after simulated transport. Food Chem..

[30] Gong M., Zhang T., Wu Y., Shang J., Su E., Cao Y., Zhang J. (2025). Synergizing postharvest physiology and nanopackaging for edible mushroom preservation. Food Chem..

[31] Sang Y., Yang W., Liu Y., Zhang W., Guo T., Shen P., Tang Y., Guo M., Chen G. (2022). Influences of low temperature on the postharvest quality and antioxidant capacity of winter jujube (*Zizyphus jujuba* Mill. cv. Dongzao). LWT.

[32] Chen M., Gu H., Wang L., Shao Y., Li R., Li W. (2022). Exogenous Ethylene Promotes Peel Color Transformation by Regulating the Degradation of Chlorophyll and Synthesis of Anthocyanin in Postharvest Mango Fruit. Front. Nutr..

[33] Zhang F., Jiang S., Jia S., Gui B., Wei Y., Chen Y., Ye J., Xu F., Ding P., Shao X. (2025). Broccoli stem extract enhances browning inhibition in fresh-cut peach fruit by inhibiting polyphenol oxidase activity and improving antioxidant capacity. Postharvest Biol. Technol..

[34] Raffo A., Moneta E., Ferrari Nicoli S., Paoletti F. (2020). GC-olfactometric characterisation of off-odours in commercially packaged rocket leaves. Food Packag. Shelf Life.

[35] Lv H., Guo S., Wu Z., Nan X., Zhu M., Mao K. (2024). Postharvest quality and metabolism changes of daylily flower buds treated with hydrogen sulfide during storage. Postharvest Biol. Technol..

[36] Bai X.-Y., Yang Z.-M., Shen W.-J., Shao Y.-Z., Zeng J.-K., Li W. (2022). Polyphenol treatment delays the browning of litchi pericarps and promotes the total antioxidant capacity of litchi fruit. Sci. Hortic..

[37] Chen L., Zhou Y., He Z., Liu Q., Lai S., Yang H. (2018). Effect of exogenous ATP on the postharvest properties and pectin degradation of mung bean sprouts (*Vigna radiata*). Food Chem..

[38] Truong V.-L., Jeong W.-S. (2021). Cellular Defensive Mechanisms of Tea Polyphenols: Structure-Activity Relationship. Int. J. Mol. Sci..

[39] Wang H.J., An D.S., Lee D.S. (2016). Development of Multifunctional Active Film and Its Application in Modified Atmosphere Packaging of Shiitake Mushrooms. J. Food Prot..

[40] Serna-Escolano V., Martínez-Romero D., Giménez M.J., Serrano M., García-Martínez S., Valero D., Valverde J.M., Zapata P.J. (2021). Enhancing antioxidant systems by preharvest treatments with methyl jasmonate and salicylic acid leads to maintain lemon quality during cold storage. Food Chem..

[41] Zhang L., Li S., Dong Y., Zhi H., Zong W. (2016). Tea polyphenols incorporated into alginate-based edible coating for quality maintenance of Chinese winter jujube under ambient temperature. LWT.

[42] Islam A., Acıkalın R., Ozturk B., Aglar E., Kaiser C. (2022). Combined effects of Aloe vera gel and modified atmosphere packaging treatments on fruit quality traits and bioactive compounds of jujube (*Ziziphus jujuba* Mill.) fruit during cold storage and shelf life. Postharvest Biol. Technol..

